# 2,4-Dihydroxy­benzaldehyde 4-ethyl­thio­semicarbazone

**DOI:** 10.1107/S160053680803300X

**Published:** 2008-10-18

**Authors:** Kong Wai Tan, Chew Hee Ng, Mohd Jamil Maah, Seik Weng Ng

**Affiliations:** a Department of Chemistry, University of Malaya, 50603 Kuala Lumpur, Malaysia; b Faculty of Engineering and Science, Universiti Tunku Abdul Rahman, 53300 Kuala Lumpur, Malaysia

## Abstract

The mol­ecular conformation of the title compound, C_10_H_13_N_3_O_2_S, is stabilized by an intramolecular O—H⋯N hydrogen bond. Adjacent mol­ecules are linked by O—H⋯O hydrogen bonds to furnish a zigzag chain.

## Related literature

For the structure of 3,4-dihydroxy­benzaldehyde 4-ethyl­thio­semicarbazone, see: Kayed *et al.* (2008 ▶).
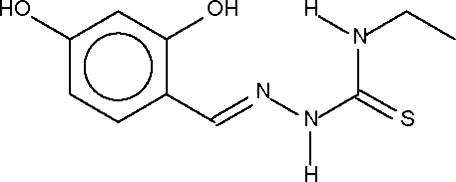


## Experimental

### 

#### Crystal data

C_10_H_13_N_3_O_2_S
*M* *_r_* = 239.29
Monoclinic, 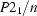

*a* = 4.6592 (6) Å
*b* = 24.067 (3) Å
*c* = 10.047 (1) Å
β = 99.060 (2)°
*V* = 1112.5 (2) Å^3^
*Z* = 4
Mo *K*α radiation
μ = 0.28 mm^−1^
*T* = 100 (2) K
0.40 × 0.12 × 0.06 mm

#### Data collection

Bruker SMART APEX diffractometer
Absorption correction: multi-scan (*SADABS*; Sheldrick, 1996 ▶) *T*_min_ = 0.896, *T*_max_ = 0.983
6303 measured reflections
2517 independent reflections
1972 reflections with *I* > 2σ(*I*)
*R*_int_ = 0.028

#### Refinement

*R*[*F* ^2^ > 2σ(*F* ^2^)] = 0.039
*wR*(*F* ^2^) = 0.109
*S* = 1.08
2517 reflections
148 parameters
H-atom parameters constrained
Δρ_max_ = 0.38 e Å^−3^
Δρ_min_ = −0.25 e Å^−3^

### 

Data collection: *APEX2* (Bruker, 2007 ▶); cell refinement: *SAINT* (Bruker, 2007 ▶); data reduction: *SAINT*; program(s) used to solve structure: *SHELXS97* (Sheldrick, 2008 ▶); program(s) used to refine structure: *SHELXL97* (Sheldrick, 2008 ▶); molecular graphics: *X-SEED* (Barbour, 2001 ▶); software used to prepare material for publication: *publCIF* (Westrip, 2008 ▶).

## Supplementary Material

Crystal structure: contains datablocks I, global. DOI: 10.1107/S160053680803300X/bt2806sup1.cif

Structure factors: contains datablocks I. DOI: 10.1107/S160053680803300X/bt2806Isup2.hkl

Additional supplementary materials:  crystallographic information; 3D view; checkCIF report

## Figures and Tables

**Table 1:** Hydrogen-bond geometry (Å, °)

*D*—H⋯*A*	*D*—H	H⋯*A*	*D*⋯*A*	*D*—H⋯*A*
O1—H1⋯N3	0.84	1.84	2.583 (2)	147
O2—H2⋯O1^i^	0.84	1.92	2.714 (2)	158

Symmetry code: (i) 
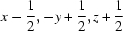
.

**Fig. 1.**
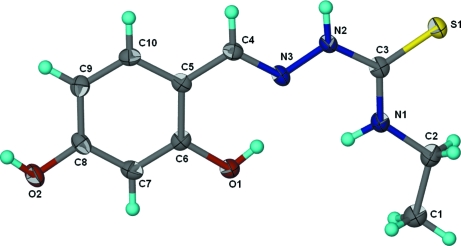
Displacement ellipsoid (Barbour, 2001) plot of the title compound at the 70% probability level. H atoms are drawn as spheres of arbitrary radius.

## supplementary crystallographic information

### Experimental

4-Ethylthiosemicarbazide (1.19 g, 10 mmol) and 2,4-dihydroxybenzaldehyde (1.38 g, 10 mmol) were refluxed in ethanol (40 ml) for 6 h. Slow evaporation of the solvent yielded yellow crystals.

### Refinement

H-atoms were placed in calculated positions (C—H 0.95 Å, N—H 0.88 Å, O—H 0.85 Å) and were included in the refinement in the riding model approximation, with *U*(H) set to 1.2*U*(C,N) or *U*(H) set to 1.5*U*(O).

### Crystal data

**Table:**

C_10_H_13_N_3_O_2_S	*F*(000) = 504
*M**_r_* = 239.29	*D*_x_ = 1.429 Mg m^−^^3^
Monoclinic, *P*2_1_/*n*	Mo *K*α radiation, λ = 0.71073 Å
Hall symbol: -P 2yn	Cell parameters from 1634 reflections
*a* = 4.6592 (6) Å	θ = 2.6–28.1°
*b* = 24.067 (3) Å	µ = 0.28 mm^−^^1^
*c* = 10.047 (1) Å	*T* = 100 K
β = 99.060 (2)°	Plate, yellow
*V* = 1112.5 (2) Å^3^	0.40 × 0.12 × 0.06 mm
*Z* = 4	

### Data collection

**Table:**

Bruker SMART APEX diffractometer	2517 independent reflections
Radiation source: fine-focus sealed tube	1972 reflections with *I* > 2σ(*I*)
graphite	*R*_int_ = 0.028
ω scans	θ_max_ = 27.5°, θ_min_ = 1.7°
Absorption correction: multi-scan (SADABS; Sheldrick, 1996)	*h* = −6→4
*T*_min_ = 0.896, *T*_max_ = 0.983	*k* = −30→31
6303 measured reflections	*l* = −13→12

### Refinement

**Table:**

Refinement on *F*^2^	Primary atom site location: structure-invariant direct methods
Least-squares matrix: full	Secondary atom site location: difference Fourier map
*R*[*F*^2^ > 2σ(*F*^2^)] = 0.039	Hydrogen site location: inferred from neighbouring sites
*wR*(*F*^2^) = 0.109	H-atom parameters constrained
*S* = 1.08	*w* = 1/[σ^2^(*F*_o_^2^) + (0.0512*P*)^2^ + 0.3651*P*] where *P* = (*F*_o_^2^ + 2*F*_c_^2^)/3
2517 reflections	(Δ/σ)_max_ = 0.001
148 parameters	Δρ_max_ = 0.38 e Å^−^^3^
0 restraints	Δρ_min_ = −0.25 e Å^−^^3^

### Fractional atomic coordinates and isotropic or equivalent isotropic displacement parameters (Å^2^)

**Table:**

	*x*	*y*	*z*	*U*_iso_*/*U*_eq_	
S1	1.01421 (11)	0.514276 (19)	0.28484 (5)	0.02112 (15)	
O1	0.1480 (3)	0.32859 (6)	0.28304 (13)	0.0240 (3)	
H1	0.2707	0.3544	0.2908	0.036*	
O2	−0.4004 (3)	0.21979 (5)	0.53651 (13)	0.0238 (3)	
H2	−0.4152	0.2117	0.6165	0.036*	
N1	0.6825 (4)	0.43087 (7)	0.17727 (16)	0.0225 (4)	
H1N	0.5495	0.4056	0.1845	0.027*	
N2	0.6791 (3)	0.44770 (6)	0.40159 (15)	0.0175 (3)	
H2N	0.7378	0.4666	0.4760	0.021*	
N3	0.4854 (3)	0.40468 (6)	0.40248 (15)	0.0169 (3)	
C1	0.6265 (5)	0.39432 (9)	−0.0504 (2)	0.0270 (5)	
H1A	0.6985	0.3971	−0.1367	0.040*	
H1B	0.4169	0.4015	−0.0644	0.040*	
H1C	0.6644	0.3569	−0.0132	0.040*	
C2	0.7804 (5)	0.43673 (9)	0.0471 (2)	0.0276 (5)	
H2A	0.9932	0.4309	0.0578	0.033*	
H2B	0.7371	0.4747	0.0115	0.033*	
C3	0.7800 (4)	0.46113 (7)	0.28620 (18)	0.0174 (4)	
C4	0.3905 (4)	0.39384 (7)	0.51361 (17)	0.0162 (4)	
H4	0.4568	0.4152	0.5920	0.019*	
C5	0.1834 (4)	0.34936 (7)	0.51966 (18)	0.0154 (4)	
C6	0.0690 (4)	0.31772 (7)	0.40586 (18)	0.0174 (4)	
C7	−0.1245 (4)	0.27525 (8)	0.41357 (19)	0.0195 (4)	
H7	−0.1983	0.2545	0.3352	0.023*	
C8	−0.2121 (4)	0.26265 (7)	0.53563 (18)	0.0179 (4)	
C9	−0.1092 (4)	0.29412 (7)	0.65039 (18)	0.0181 (4)	
H9	−0.1729	0.2864	0.7338	0.022*	
C10	0.0857 (4)	0.33653 (7)	0.64102 (18)	0.0171 (4)	
H10	0.1560	0.3577	0.7192	0.021*	

### Atomic displacement parameters (Å^2^)

**Table:**

	*U*^11^	*U*^22^	*U*^33^	*U*^12^	*U*^13^	*U*^23^
S1	0.0235 (3)	0.0188 (2)	0.0214 (2)	−0.0054 (2)	0.00430 (19)	0.00073 (18)
O1	0.0316 (9)	0.0240 (7)	0.0169 (6)	−0.0099 (6)	0.0059 (6)	−0.0026 (5)
O2	0.0275 (8)	0.0207 (7)	0.0225 (7)	−0.0089 (6)	0.0019 (6)	0.0036 (5)
N1	0.0235 (9)	0.0242 (8)	0.0207 (8)	−0.0087 (7)	0.0063 (7)	−0.0028 (7)
N2	0.0181 (8)	0.0176 (8)	0.0167 (7)	−0.0047 (6)	0.0022 (6)	−0.0009 (6)
N3	0.0154 (8)	0.0139 (7)	0.0211 (8)	−0.0016 (6)	0.0019 (6)	0.0017 (6)
C1	0.0302 (12)	0.0283 (11)	0.0228 (10)	−0.0037 (9)	0.0056 (9)	−0.0022 (8)
C2	0.0301 (12)	0.0332 (11)	0.0209 (10)	−0.0089 (10)	0.0081 (9)	−0.0025 (8)
C3	0.0141 (10)	0.0168 (9)	0.0209 (9)	0.0029 (7)	0.0017 (7)	0.0019 (7)
C4	0.0151 (10)	0.0162 (8)	0.0165 (9)	0.0004 (7)	0.0004 (7)	−0.0011 (7)
C5	0.0138 (9)	0.0133 (8)	0.0185 (9)	0.0021 (7)	0.0006 (7)	0.0002 (7)
C6	0.0176 (10)	0.0179 (9)	0.0166 (9)	0.0025 (7)	0.0027 (7)	0.0007 (7)
C7	0.0208 (10)	0.0169 (9)	0.0195 (9)	−0.0012 (8)	−0.0010 (8)	−0.0014 (7)
C8	0.0154 (10)	0.0139 (8)	0.0232 (9)	−0.0007 (7)	−0.0005 (8)	0.0031 (7)
C9	0.0184 (10)	0.0190 (9)	0.0168 (9)	0.0012 (7)	0.0021 (7)	0.0033 (7)
C10	0.0180 (10)	0.0170 (9)	0.0155 (8)	0.0015 (8)	−0.0003 (7)	−0.0017 (7)

### Geometric parameters (Å, °)

**Table:**

S1—C3	1.6826 (19)	C1—H1C	0.9800
O1—C6	1.367 (2)	C2—H2A	0.9900
O1—H1	0.8400	C2—H2B	0.9900
O2—C8	1.355 (2)	C4—C5	1.449 (2)
O2—H2	0.8400	C4—H4	0.9500
N1—C3	1.333 (2)	C5—C10	1.401 (2)
N1—C2	1.458 (2)	C5—C6	1.407 (2)
N1—H1N	0.8800	C6—C7	1.373 (3)
N2—C3	1.357 (2)	C7—C8	1.386 (3)
N2—N3	1.374 (2)	C7—H7	0.9500
N2—H2N	0.8800	C8—C9	1.400 (3)
N3—C4	1.291 (2)	C9—C10	1.379 (3)
C1—C2	1.515 (3)	C9—H9	0.9500
C1—H1A	0.9800	C10—H10	0.9500
C1—H1B	0.9800		
			
C6—O1—H1	109.5	N2—C3—S1	120.03 (14)
C8—O2—H2	109.5	N3—C4—C5	120.45 (16)
C3—N1—C2	124.66 (16)	N3—C4—H4	119.8
C3—N1—H1N	117.7	C5—C4—H4	119.8
C2—N1—H1N	117.7	C10—C5—C6	117.04 (17)
C3—N2—N3	120.08 (15)	C10—C5—C4	120.59 (16)
C3—N2—H2N	120.0	C6—C5—C4	122.37 (16)
N3—N2—H2N	120.0	O1—C6—C7	117.76 (17)
C4—N3—N2	118.21 (15)	O1—C6—C5	120.57 (17)
C2—C1—H1A	109.5	C7—C6—C5	121.67 (17)
C2—C1—H1B	109.5	C6—C7—C8	120.03 (17)
H1A—C1—H1B	109.5	C6—C7—H7	120.0
C2—C1—H1C	109.5	C8—C7—H7	120.0
H1A—C1—H1C	109.5	O2—C8—C7	116.98 (17)
H1B—C1—H1C	109.5	O2—C8—C9	123.01 (17)
N1—C2—C1	109.36 (17)	C7—C8—C9	120.00 (17)
N1—C2—H2A	109.8	C10—C9—C8	119.21 (17)
C1—C2—H2A	109.8	C10—C9—H9	120.4
N1—C2—H2B	109.8	C8—C9—H9	120.4
C1—C2—H2B	109.8	C9—C10—C5	122.00 (17)
H2A—C2—H2B	108.3	C9—C10—H10	119.0
N1—C3—N2	116.86 (17)	C5—C10—H10	119.0
N1—C3—S1	123.11 (14)		
			
C3—N2—N3—C4	−178.76 (17)	C10—C5—C6—C7	−1.5 (3)
C3—N1—C2—C1	178.52 (18)	C4—C5—C6—C7	179.20 (17)
C2—N1—C3—N2	−175.78 (18)	O1—C6—C7—C8	−179.83 (17)
C2—N1—C3—S1	4.9 (3)	C5—C6—C7—C8	0.1 (3)
N3—N2—C3—N1	0.0 (2)	C6—C7—C8—O2	−179.15 (16)
N3—N2—C3—S1	179.30 (13)	C6—C7—C8—C9	1.6 (3)
N2—N3—C4—C5	179.27 (15)	O2—C8—C9—C10	179.01 (17)
N3—C4—C5—C10	178.30 (17)	C7—C8—C9—C10	−1.8 (3)
N3—C4—C5—C6	−2.4 (3)	C8—C9—C10—C5	0.3 (3)
C10—C5—C6—O1	178.39 (16)	C6—C5—C10—C9	1.3 (3)
C4—C5—C6—O1	−0.9 (3)	C4—C5—C10—C9	−179.39 (17)

### Hydrogen-bond geometry (Å, °)

**Table:**

*D*—H···*A*	*D*—H	H···*A*	*D*···*A*	*D*—H···*A*
O1—H1···N3	0.84	1.84	2.583 (2)	147
O2—H2···O1^i^	0.84	1.92	2.714 (2)	158

Symmetry codes: (i) *x*−1/2, −*y*+1/2, *z*+1/2.
